# Ultra‐Fast Self‐Powered Heterojunction Blue‐Light Photodetector Based on Boronate‐Ester‐Linked COF‐5

**DOI:** 10.1002/anie.202502364

**Published:** 2025-07-17

**Authors:** Shuangyin Gao, Rujun Tang, Ping Duan, Jin Tan, Shuoguo Yuan, Zhigao Dai, Jianmei Xu, Zhihong Yang, Wei Zhou, Auttaphon Chachvalvutikul, Kieran Aggett, Anatoly Zayats, Ouardia Akdim, Jian Sun, Graham Hutchings

**Affiliations:** ^1^ Faculty of Materials Science and Chemistry China University of Geosciences Wuhan 430074 P.R. China; ^2^ Jiangsu Key Laboratory of Thin Films, School of Physical Science and Technology Soochow University Suzhou 215123 China; ^3^ Department of Physics and London Centre for Nanotechnology King's College London London WS2R 2LS UK; ^4^ Max Planck‐Cardiff Centre on the Fundamentals of Heterogeneous Catalysis FUNCAT, Cardiff Catalysis Institute, School of Chemistry Cardiff University Translational Research Hub Cardiff CF24 4HQ UK

**Keywords:** 2D‐COF, Heterojunction, Optoelectronic, Self‐powered blue‐photodetectors

## Abstract

Covalent organic framework materials have recently garnered significant interest from the scientific community due to their fascinating properties that include highly ordered porosity, structural versatility, high chemical and thermal stabilities, and facile surface modification. Herein, for the first time, we present the design and fabrication of a self‐powered blue‐light photodetector based on boronate‐ester‐linked 2D covalent organic framework (COF‐5) film, synthesized using hexahydroxytriphenylene and 1,4‐phenylenediboronic acid organic linkers. Specifically, we have developed a COF‐5/n‐Si photodetector that exhibits an ultra‐short rise response time of 41 µs and a decay response time of 222 µs under 0 V bias. Our work integrated rapid response times and the distinctive benefit of self‐powering, setting it apart from existing COF‐based photodetectors.

## Graphical abstract

We report the design and fabrication of a self‐powered blue‐light photodetector based on boronate‐ester‐linked 2D covalent organic framework (COF‐5) film, synthesized using hexahydroxytriphenylene and 1,4‐phenylenediboronic acid organic linkers. The p‐COF‐5/n‐Si photodetector exhibits an ultra‐short rise time of 41 µs and a decay time of 222 µs under 0 V bias.

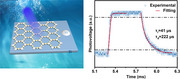

## Introduction

Since Yaghi's pioneering report in 2005,^[^ ^1^ ^]^ covalent organic frameworks (COFs) have garnered worldwide interest owing to their exceptional properties, including high thermochemical stability, low density, large surface area, tunable physicochemical characteristics, versatile molecular architectures, and diversity in synthesis strategies.^[^ ^2^, ^3^ ^]^ Furthermore, inspired by the remarkable electronic and structural properties of graphene, the quest for 2D π‐conjugated COFs has surged, driven by considerable expectations.^[^ ^4^ ^]^ Indeed, 2D π‐conjugated COFs, a class of organic crystalline materials with predesigned π‐electronic skeletons and topological structures, are covalently constructed from 2D building blocks. These building blocks are arranged into periodic planar networks and are stacked to form layered structures. These properties confer them rapid charge carrier transport, enabling their application in optoelectronics.^[^ ^5^, ^6^ ^]^ Albeit their intrinsically low exciton binding energy,^[^ ^7^ ^]^ investigations of COFs in photodetector application remain scarce to date. For example, in 2020, Xiong et al. reported a COF_ETBC–TAPT_‐graphene heterostructure‐based photodetector exhibiting a time response of ca. 1.14 ms at 1 V.^[^ ^8^ ^]^ In 2023, Bag et al. constructed a photodetector with a glass/FTO/TiO₂/COF‐film/Au architecture, utilizing a TpEtBr COF thin film (Tp: trialdehyde; EtBr: 5‐ethyl‐6‐phenylphenanthridine‐3,8‐diamine bromide). The device achieved a photocurrent density of 2.65 ± 0.24 mA cm⁻^2^ at 0.5 V; however, the study did not provide any information regarding the response time.^[^ ^9^ ^]^ Recently, Gu et al. and Tang et al. employed chiral COFs as the active layers of photodetectors to detect circularly polarized light. Under a 1 V bias, Tang's D‐Tar‐Tz photodetector exhibited response time of 596 ms/504 ms; under a 0.1 V bias, Gu's CityU‐8 COF photodetector showed response time of 35.2 ms/35.2 ms.^[^ ^10^, ^11^ ^]^ One of the main challenges of these photodetectors is that they are not self‐powered. Self‐powered photodetectors operate without the need for external power, deriving the necessary energy directly from incoming light. This self‐sustaining operation is not only energy‐efficient but also allows for reliable deployment in remote or portable devices.^[^ ^12^, ^13^ ^]^ These photodetectors typically have lower capacitance, which enables faster response times by reducing charge accumulation, which is an advantage in high‐speed applications like optical communication.^[^ ^14^, ^15^ ^]^ Furthermore, they exhibit minimal dark current, resulting in a high signal‐to‐noise ratio (SNR). This low dark current and improved SNR significantly enhance sensitivity, especially in low‐light conditions, enabling accurate detection of weak signals in environments such as night vision.^[^ ^16^, ^17^, ^18^ ^]^ Altogether, self‐powered photodetectors offer a unique combination of efficiency, speed, and sensitivity that makes them highly valuable across a wide range of optical sensing applications.

Herein, we pioneer the development of a self‐powered blue‐light photodetector by fabricating a boronate‐ester‐linked COF‐5/n‐Si heterojunction structure, which operates at ultra‐high speed. The COF‐5/n‐Si photodetector exhibits an ultra‐short rise time of 41 µs, a decay time of 222 µs at 0 V bias, demonstrating the highest performance compared to current COF‐based photodetectors. These characteristics position our material as a promising candidate for photodetection applications.^[^ ^19^ ^]^

## Results and Discussion

**Figure 1:**
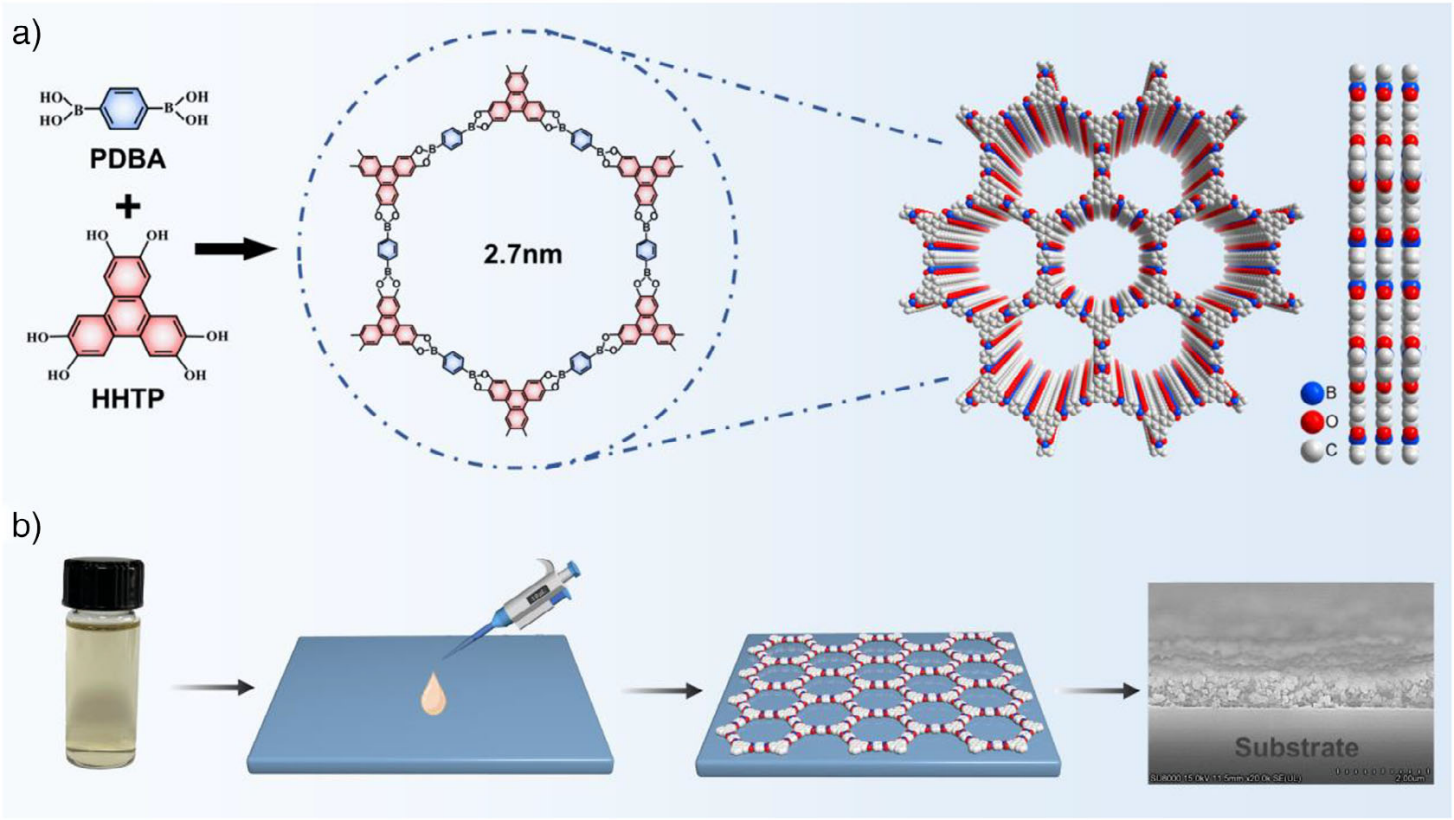
a) Reaction scheme for boronate‐ester‐linked COF‐5 colloidal ink formation. b) Fabrication of COF‐5 film through drop‐casting and scanning electron microscopy image of the COF‐5 film.

**Figure 2:**
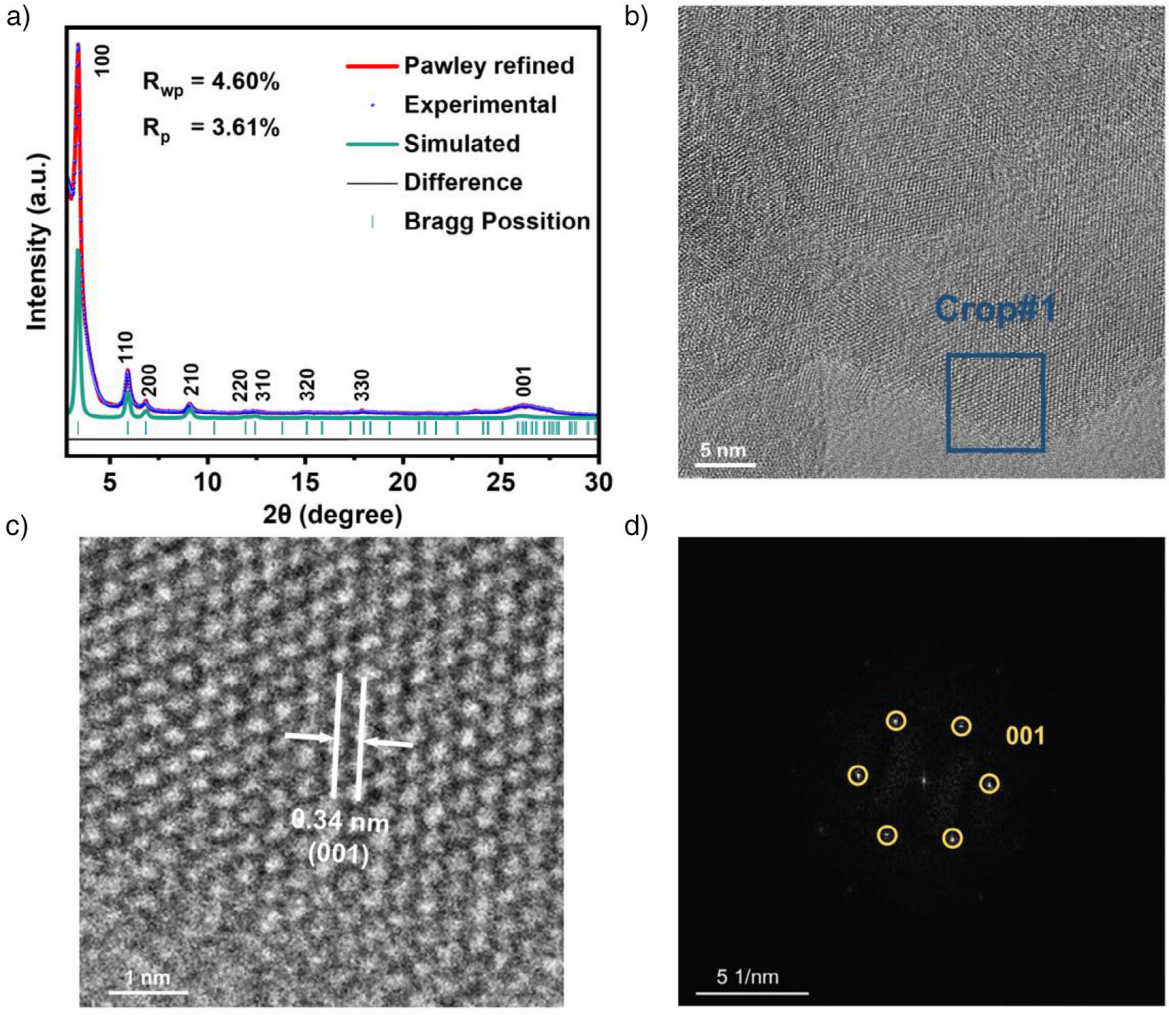
a) XRD spectrum of COF‐5 thin film. b) and c) High‐resolution transmission electron microscopy (TEM) images of the COF‐5. d) Fast Fourier transform derived from the high‐resolution TEM images.

Boronate‐ester‐linked COF‐5 colloidal solution was synthesized by condensation of hexahydroxytriphenylene (HHTP) and 1,4‐phenylenediboronic acid (PDBA) organic linkers, using a solvothermal method (Figure 1a; see Supporting Information for synthesis details). The structure consists of a highly ordered, periodic hexagonal structure, with each hexagonal unit of 2.7 nm made up of alternating boron (B), oxygen (O), and carbon (C) atoms, creating a repeating and symmetric pattern. The framework is held together by boronic ester linkages, which are formed through the condensation reaction between boronic acid groups (─B(OH)_₂_) on PDBA and hydroxyl groups (─OH) on HHTP. The resulting COF‐5 colloid displays an average particle diameter of 30 nm (Figure S1), enabling the particles to remain relatively stable within the colloidal suspension.^[^ ^20^ ^]^ To assess the COF film's potential for thin‐film optoelectronic devices, the 2D COF‐5 films were fabricated via a drop‐casting method (Figure 1b and Supporting Information) that is recognized for its rapidity, versatile patterning, and scalability.^[^ ^21^ ^]^ Scanning electron microscopy of the film's cross‐section demonstrates a network‐like framework structure (Figure 1b). Atomic force microscopy (AFM) revealed the successful continuous and smooth covalent coverage of the glass slide by the COF‐5 film, with an average surface roughness of 15.8 nm (Figure S2), consistent with Li et al.’s work.^[^ ^22^ ^]^ The X‐ray diffraction (XRD) patterns divulge nine distinct peaks at 2*θ* values of 3.39°, 5.93°, 6.90°, 9.11°, 11.9°, 12.4°, 15.07°, 17.9°, and 26.6°, which are attributed to the (100), (110), (200), (210), (220), (310), (320), (330), and (001) crystal planes of COF‐5, respectively.^[^ ^23^ ^]^ Structural modeling and optimization of an AA‐stacked hexagonal COF‐5 structural model using Materials Studio yielded lattice parameters of *a* = *b* = 29.706 Å, *c* = 3.4607 Å, and *α* = *β* = 90°, *γ* = 120° (space group *P*6/*m*). Subsequent Pawley refinement of the PXRD data, based on this AA‐stacked model, demonstrated excellent agreement between the experimental and simulated patterns, with minimal fitting errors (*R*_wp_ = 4.60% and *R*_p_ = 3.61%). The high‐resolution transmission electron microscopy (HR‐TEM) images (Figure 2b,c) further confirm the high crystallinity of COF‐5, prominently displaying lattice fringes corresponding to the (001) crystal plane, with an interplanar spacing of ca. 0.34 nm.^[^ ^24^, ^25^ ^]^ The successful formation of borate─ester bonds within the COF‐5 colloid was confirmed by Fourier transform infrared spectroscopy (FTIR) (Figure S3a), which displays the infrared characteristic vibrational peaks at 1334, 1242, and 1017 cm^−1^, corresponding to the stretching vibrations of B─O, C─O, and B─C bonds, respectively.^[^ ^26^ ^]^ The thermal stability of the material was assessed by thermogravimetry (Figure S3b), and indicated a strong stability of the COF‐5 until 90 °C, ensuring that the inherent structure remains intact during further film fabrication and performance testing.^[^ ^27^ ^]^ In addition, the COF‐5 film exhibited a large surface area of 1956 m^2^g^−1^ (Figure S3c), that can effectively facilitate the photons absorption.^[^ ^28^ ^]^

**Figure 3:**
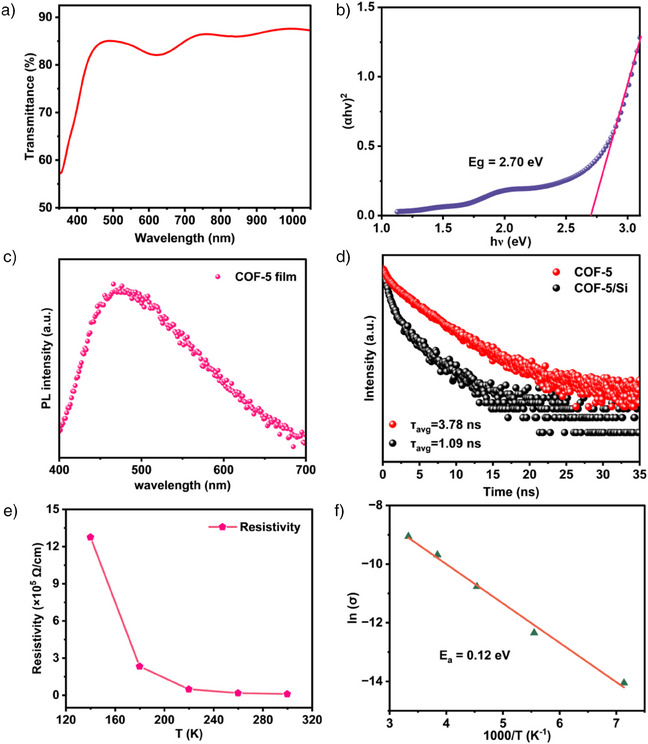
a) UV–vis transmission spectrum of the COF‐5 film. b) Tauc plot of the COF‐5 film. c) Photoluminescence spectrum of the COF‐5 film with an excitation wavelength of 375 nm. d) Time‐resolved fluorescence spectra of the COF‐5 film and of the COF‐5/Si heterojunction at an excitation wavelength of 375 nm. e) Resistivity of the COF‐5 film as a function of temperature ranging from 140 to 300 K. f) Plot of ln(*σ*) versus *T*⁻¹.

Subsequently, the optical and electronic properties of the materials were systematically evaluated. The COF‐5 film exhibited an average transmittance of 81% in the visible light range (400–800 nm), demonstrating high transparency (Figure 3a). The variation in transmittance observed around 500–700 nm suggests an interaction between light and the electronic structure of the COF framework. This behavior can be attributed to π–π* transitions within the aromatic HHTP core and resonance effects associated with the boronate ester linkages. Besides, the wide band gap of 2.7 eV, confirmed by the photoluminescence (PL) peak at 460 nm, enables the photodetector to efficiently absorb and detect blue light (Figure 3b,c).^[^ ^29^, ^30^ ^]^ Time‐resolved photoluminescence (TRPL) spectroscopy measurements (Figure 3d) were performed on COF‐5 films deposited on both n‐Si and glass substrates, using a 460 nm pulsed light excitation. The charge carrier lifetime at the p‐COF‐5 film is *τ* = 3.78 ns. Figure 3e depicts the temperature dependence of the resistivity of the COF‐5 film, which decreases as the temperature increases, demonstrating typical semiconducting behavior.^[^ ^31^ ^]^ This trend indicates that the material's conductivity improves with temperature, consistent with enhanced charge mobility. In Figure 3f, the fitted activation energy for the COF‐5 film is ca. 0.12 eV, which is significantly lower than the 0.21 eV reported by Toda et al.^[^ ^25^ ^]^ This lower activation energy suggests a reduced energy barrier for charge transport in the COF‐5 film, which translates into superior performance.

**Figure 4:**
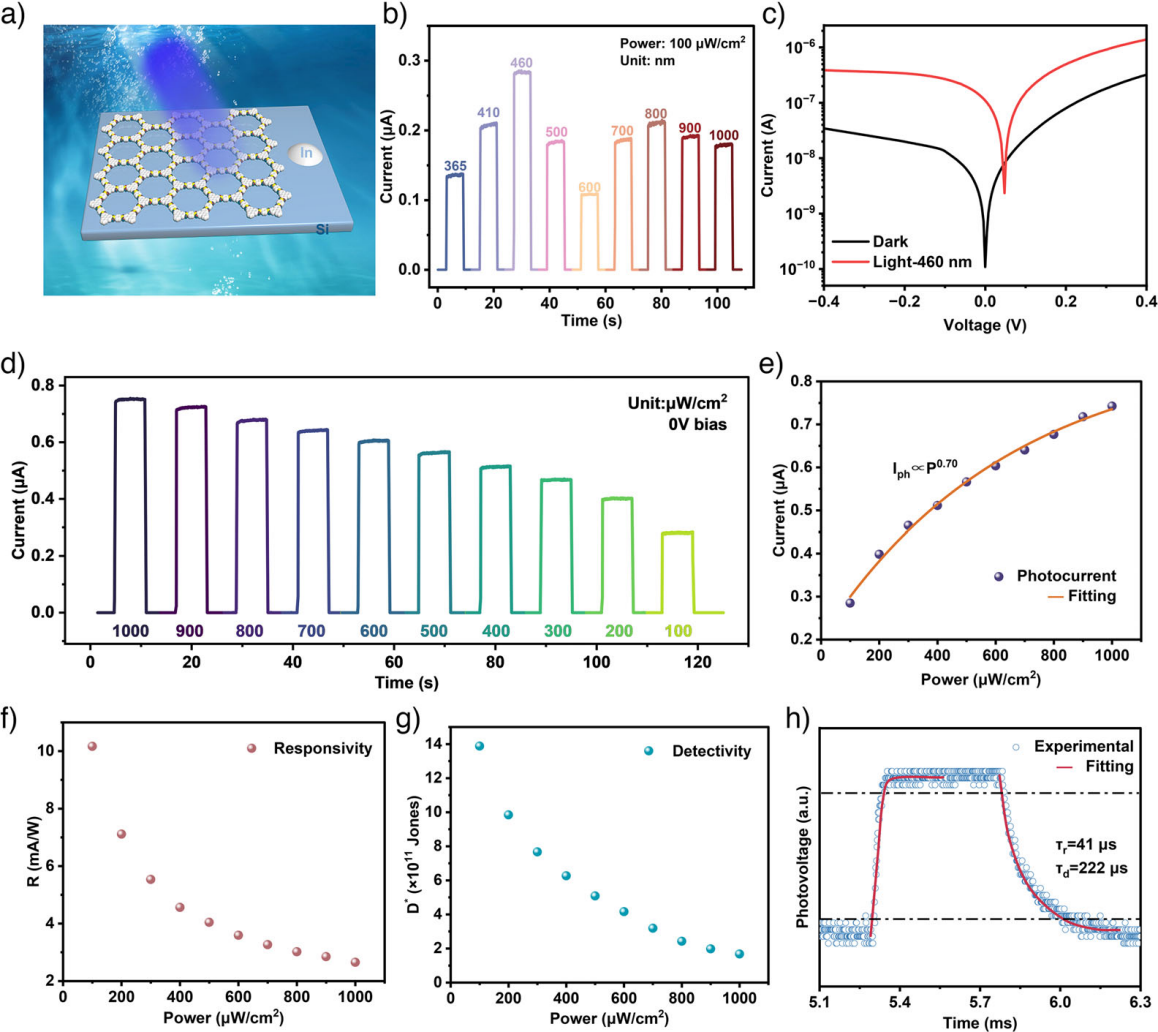
Photoelectric properties of the p‐COF‐5/n‐Si photodetector. a) Schematic diagram of the p‐COF‐5/n‐Si photodetector. b) *I*–*t* images under 0 V bias at different wavelengths of monochromatic light irradiation. c) *I*–*V* characteristic curves in the dark state and under 460 nm illumination at 100 µW cm^−2^. d) Transient response of the photodetector under differing light intensities at 460 nm illumination with 0 V bias condition. e) Correlation of photocurrent, f) responsivity, g) detectivity with optical power density, and h) response time plot of the photodetector under 0 V bias and 460 nm monochromatic illumination.

Building upon the good optical and electrical properties of the COF‐5 film, a p‐COF‐5/n‐Si heterojunction self‐powered photodetector was fabricated, and the device structure is schematically displayed in Figure 4a. The charge carrier lifetime at the p‐COF‐5 film/n‐Si heterojunction interface (*τ* = 1.09 ns) was substantially shorter compared to that of the COF‐5 film/glass substrate (Figure 3d) suggesting a facilitated carrier transfer and a strong coupling effect at the COF‐5 film/Si interface.^[^ ^32^ ^]^ The transient absorption (TA) spectra of COF‐5/Si are shown in Figure S4. The TA decay kinetic trace at ∼721 nm of our COF‐5/Si in Figure S4(c) obviously exhibits faster decay rate than the TA decay kinetic trace of COF‐5 reported by Nathan et al.,^[^ ^33^ ^]^ matching well with the above PL result. This demonstrates enhanced charge transport characteristics at the COF‐5 film/Si interface, suggesting the formation of a favorable band alignment between p‐type COF‐5 and n‐type Si, simultaneously leading to an effective built‐in electric field that promotes spontaneous photogenerated carrier separation without the need for external bias. In the aim to evaluate the spectral photo‐response characteristics, wavelength‐dependent photocurrent measurements were conducted under monochromatic illumination spanning from ultraviolet (365 nm) to near‐infrared (1000 nm), at a constant incident power density of 100 µW cm^−^^2^ (Figure 4b). The photocurrent response curves were acquired to assess the device's spectral sensitivity and response dynamics. The photocurrent under 460 nm stands out as the highest across the entire wavelength range. This peak response at 460 nm is due to the close match between the photon energy (ca. 2.7 eV) and the COF‐5′s bandgap energy. At this wavelength, the incoming photons possess sufficient energy to efficiently excite electrons from the valence band to the conduction band, generating electron‐hole pairs effectively. Furthermore, the lower recombination rate of electron‐hole pairs under these conditions contributes to a higher photocurrent. This combination of factors underscores the device's enhanced sensitivity, affirming its potential for applications requiring precise photodetection at this specific wavelength.

Figure 4c illustrates the current–voltage (*I*–*V*) characteristics of the p‐COF‐5/n‐Si photodetector both in the absence of light (dark state) and under illumination from a 460 nm light source at an intensity of 100 µW cm^−^^2^. In the dark state, the *I*–*V* curves clearly display rectifying behavior, indicative of typical p–n junction diode characteristics. When irradiated at 460 nm, the p‐COF‐5/n‐Si photodetector exhibits a significant photoelectric response, as evidenced by a high on/off ratio of 1.8 × 10^3^. The substantial enhancement in photocurrent, under illumination, demonstrates the photodetector's efficient optoelectronic conversion capability, further highlighting its potential for high‐sensitivity photodetection applications. The intensity‐dependent photoresponse characteristics of the p‐COF‐5/n‐Si heterojunction photodetector were evaluated under zero‐bias conditions (Figure 4d). Time‐resolved photocurrent measurements were conducted under 460 nm monochromatic illumination with incident power densities ranging from 100 to 1000 µW cm^−^^2^. This sequential characterization under different illumination intensities enables quantitative assessment of the detector's linear dynamic range, sensitivity threshold, and potential saturation effects, which are essential parameters for practical optoelectronic applications. The photocurrent magnitude scales proportionally with the incident light intensity, following the expected behavior of photogenerated carrier density dependence on photon flux. These characteristics, combined with stable operation without external bias, validate the device's reliability and sensitivity for self‐powered photodetection applications.

Figure 4e–g illustrate the performance characteristics of the p‐COF‐5/n‐Si photodetector, showing the relationships between photocurrent (*I*_ph_), light intensity, responsivity (*R*), and detectivity (D*) under varying illumination conditions. The relationship between photocurrent (*I*_ph_) and light intensity (*P*) can be described by Equation (1):^[^ ^34^ ^]^

(1) $$I_{\mathrm{ph}}\propto P^{\theta }$$

where *I*_ph_ represents the difference between the light current (*I*_light_) and dark current (*I*_dark_), expressed as *I*_ph_ = *I*_light_ **− *I*_dark_, and *θ* is determined by the power relationship between light intensity and photocurrent. *θ* was determined to be 0.7 via curve fitting (Figure 4e). This value deviates from the theoretical maximum of 1, indicating potential carrier recombination losses during photodetection.^[^ ^35^ ^]^ As shown in Figure 4f,g, *R*, and D* decrease with the increase of the light intensity (Equations S1 and S2). The maximum *R* and D* values of the p‐COF‐5/n‐Si photodetector reached 12 mA W^−1^ and 1.4 × 10^12^ Jones, respectively, at an incident light intensity of 100 µW cm^−2^. A 460 nm monochromatic light source was applied to stimulate the p‐COF‐5/n‐Si photodetector, and the response speed was evaluated using a digital oscilloscope. A waveform in the photoresponse was intercepted and amplified for a second‐order exponential fitting on the rise and decay processes, as shown in Figure 4h. The response rise time (*τ*_r_) and the decay time (*τ*_d_) were, respectively, 41 and 222 µs for the p‐COF‐5/n‐Si photodetector. Compared to typical p–n junction self‐powered blue‐light photodetector based on main 2D materials, the COF‐5/n‐Si blue photodetector outperforms most other typical p–n junction self‐powered blue photodetectors in terms of response speed (Table S1).

**Figure 5:**
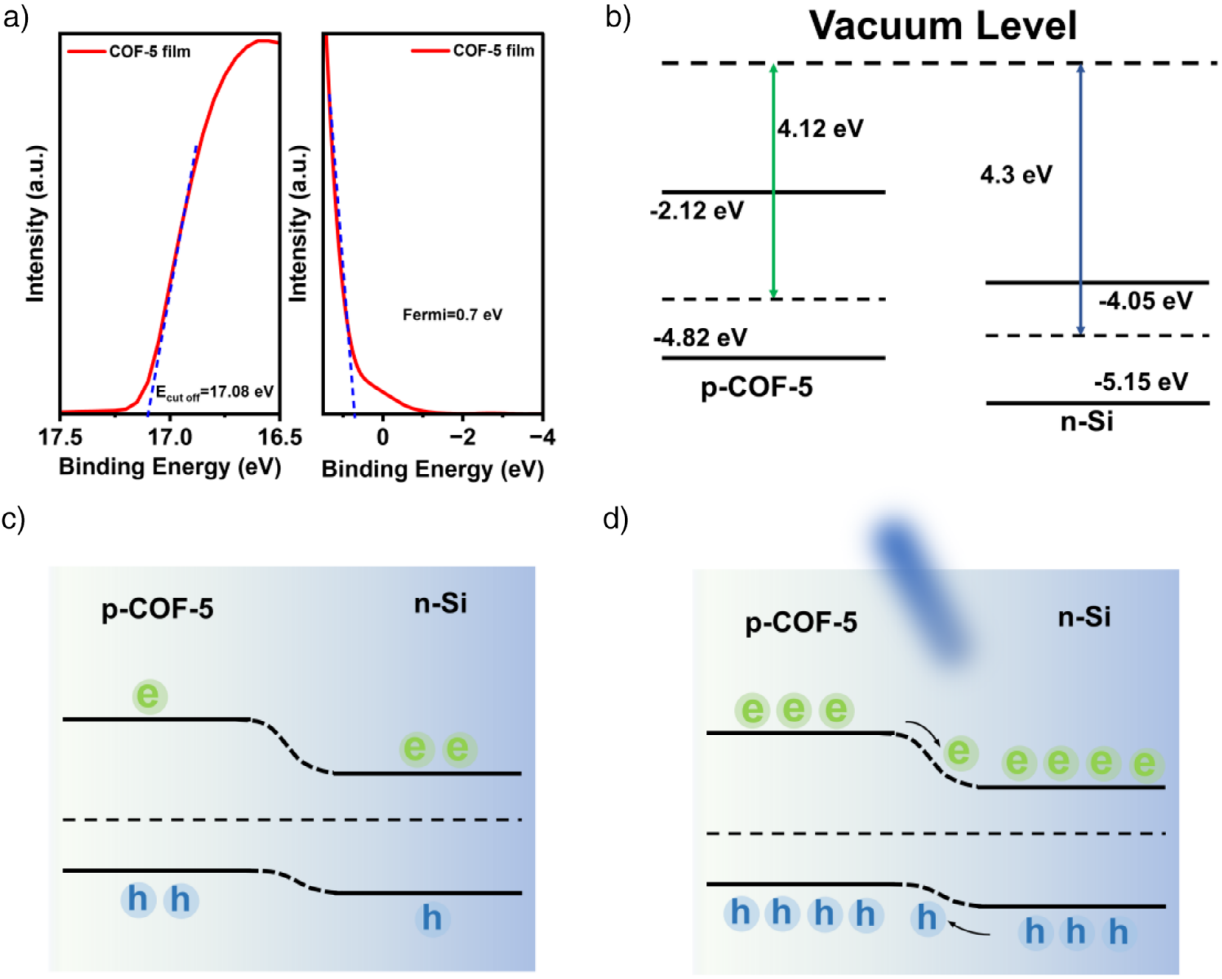
a) Ultraviolet photoelectron spectroscopy of the COF‐5 film. b)–d) Schematic diagram of the COF‐5/n‐Si band structure.

In order to understand the self‐powered mode of the p‐COF‐5/n‐Si photodetector, the Ultraviolet photoelectron spectroscopy (UPS) was carried out and the band structure was evaluated. The work function *Φ* of the COF‐5 film was calculated according to Equation S3. As shown in Figure 5a, *E*_cutoff_ (the secondary electron cutoff edge) is 17.08 eV, and the difference between the Fermi energy level and the maximum value of the valence band ((Δ*E*_COF‐5_)) is 0.70 eV, leading to a work function of 4.12 eV and a maximum valence band of −4.82 eV, Equation (S4). From Figure 3b, the energy band gap (*E*_g_) of COF‐5 is determined to be 2.7 eV, while the minimum value of the conduction band (*E*_c_) for the COF‐5 film is −2.12 eV (Equation S5). The energy band structures of COF‐5 and Si are finally shown in Figure 5b, demonstrating a type II heterostructure.^[^ ^36^ ^]^ Figure 5c,d illustrate the band diagrams of the p‐COF‐5/n‐Si heterojunction photodetector in the dark and under illumination. As shown in Figure 5c, due to the diffusion and drift motions of the intrinsic carriers, the energy bands were bent, and a depletion layer was formed at the interface. Meanwhile, a built‐in electric field pointing from the n‐Si region to the p‐COF‐5 region was formed in the depletion layer. Under 460 nm light irradiation, the photogenerated electron‐hole pairs in the depletion layer were separated by the built‐in electric field, and the electrons were transported from the conduction band of COF‐5 to the conduction band of Si, and the holes were transported from the valence band of Si to the valence band of COF‐5, as depicted in Figure 5d.^[^ ^37^, ^38^ ^]^ As a result, photogenerated carriers generate a photocurrent in the external circuit, enabling the device to achieve self‐powered optical detection.

## Conclusion

In this paper, boronate‐ester‐linked COF‐5 colloid was synthesized using hexahydroxytriphenylene (HHTP) and 1,4‐phenylenediboronic acid (PDBA) as organic linkers, and subsequently 2D thin films were coated by drop‐casting method on both glass and n‐Si substrates. A novel blue‐light photodetector was fabricated using the COF‐5/n‐Si structure. This photodetector operates in a self‐powered manner and exhibits superior performance with an ultra‐short response time of 41/222 µs, surpassing all current COF‐based photodetector and most other typical p–n junction self‐powered blue‐light photodetectors based on main 2D materials. This study highlights the potential of COF films in advancing fast‐response optoelectronics. The self‐powered design, eliminating the need for external power, makes it suitable for standalone optoelectronic and portable sensing applications.

## Conflict of Interests

The authors declare no conflict of interest.

## Supporting information



Supporting Information

## Data Availability

The data that support the findings of this study are available in the Supporting Information of this article.
