# Supplementary material for: Ultra‐Fast Self‐Powered Heterojunction Blue‐Light Photodetector Based on Boronate‐Ester‐Linked COF‐5
Source: Angew Chem Int Ed Engl. 2025 Jul 17;64(36):e202502364. doi: 10.1002/anie.202502364 (PMC12402839; doi:10.1002/anie.202502364)
Supplement: Supplementary file 1 — Supporting Information [file ANIE-64-e202502364-s001.docx]

**Ultra-Fast Self-Powered Heterojunction Blue-light Photodetector Based on Boronate-ester-linked COF-5**

Shuangyin Gao^1^, Rujun Tang^2^, Ping Duan^1^, Jin Tan^1^, Shuoguo Yuan^1^, Zhigao Dai^1^, Jianmei Xu^1^, Zhihong Yang^1^, Wei Zhou^1^, Auttaphon Chachvalvutikul^4^, Kieran Aggett^4^, Anatoly Zayats^3^, Ouardia Akdim^4*^, Jian Sun^1,4*^, Graham Hutchings^4*^

^1^ Faculty of Materials Science and Chemistry, China University of Geosciences, Wuhan, 430074, P.R. China



^2^ Jiangsu Key Laboratory of Thin Films, School of Physical Science and Technology, Soochow University, Suzhou 215123, China

^3^ Department of Physics and London Centre for Nanotechnology, King’s College London, London, WS2R 2LS, UK

^4^ Max Planck-Cardiff Centre on the Fundamentals of Heterogeneous Catalysis FUNCAT, Cardiff Catalysis Institute, School of Chemistry, Cardiff University, Translational Research Hub, Cardiff, CF24 4HQ, United Kingdom

^*^Corresponding author:

[hutch@cardiff.ac.uk](mailto:hutch@cardiff.ac.uk), [sunjian@cug.edu.cn](mailto:sunjian@cug.edu.cn), [akdimo@cardiff.ac.uk](mailto:akdimo@cardiff.ac.uk)

**Experimental section**

*Synthesis of COF-5 and the photodetector*

2,3,6,7,10,11-hexahydroxytriphenyl (HHTP, 0.02 mmol) and 1,4-phenylenediboric acid (BA, 0.03 mmol) were dissolved in a mixture of CH_3_CN: 1,4-dioxane: mesitylene (80:16:4 by volume; 10 ml), and then ultrasonically agitated. The solution was passed through a 0.45-micron PTFE syringe filter to remove insoluble particulates. The filtered solution was subsequently filtered in a scintillation bottle and sealed. The solution was then heated to 90 ℃ without stirring and reacted for 20 hours to obtain a stable and translucent COF-5 colloidal suspension. The COF-5 colloidal inks was drop-cast onto different substrates (*i.e.*, glass, n-Si, and SiO_2_/n-Si ) to form a COF-5 film. After coating onto an n-Si substrate, an Ag electrode was fabricated on the COF-5 and an In electrode was fabricated on the n-Si substrate, creating a COF-5/n-Si heterojunction photodetector.

*Physicochemical Characterization methods*

The morphology and crystal structure of the COF-5 film were characterized by scanning electron microscopy (SEM, SU8010, HITACHI, Japan), transmission electron microscopy (TEM, F200x, Thermofisher Scientific, USA), atomic force microscope (AFM, Cypher), and X-ray diffraction (XRD, AXS D8 Advance, Bruker, Germanyn) using Cu Kα radiation (λ = 1.5406 Å). The particle size of the COF-5 colloid was analyzed using a nanoparticle size analyzer (DLS, Zetasizer Nano ZS, Malvern, U.K.). Fourier-transform infrared (FTIR) spectroscopy was performed using an FTIR spectrometer (Nicolet iS50, Thermo Fisher Scientific, USA), and thermal stability was assessed by thermogravimetric analysis (TGA, TG 209 F1 Libra, Netzsch, Germany).

*Optoelectronic characterization methods*

The optical properties of the COF-5 film were obtained using a UV-Vis-near-infrared spectrophotometer (Specord 200 Plus, Analytik Jena, Germany) and a transient fluorescence spectrometer (FLS1000, Edinburgh Instruments Ltd., U.K.). Transient Absorption (TA) Spectroscopy was performed on a pump-probe system (Helios, Ultrafast System). The electrical properties of the COF-5 were evaluated using a vacuum cryogenic probe station (SKDT-200, Shenyang ShuoKe Technology Co., Ltd.) and a Hall effect tester (HALL 8800, Shanghai Feibai Technology Co., Ltd.). Ultraviolet photoelectron spectroscopy (UPS) measurements were conducted using a UPS system (PHI 5000 VersaProbe, ULVAC-PHI, Japan), equipped with a He-I (21.2 eV) discharge lamp. The COF-5 based photodetector's performance was tested by a semiconductor parameter analyzer (Keithley 4200-SCS), and the according response speed was assessed using a Tektronix DPO5140B digital oscilloscope.


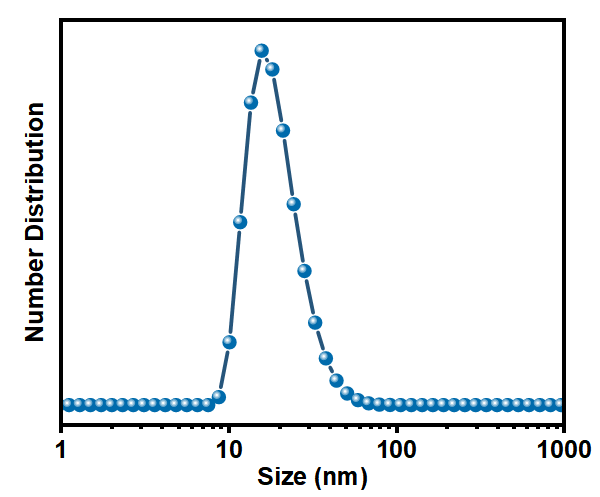


Figure S1. DLS particle size distribution of the COF-5 colloid


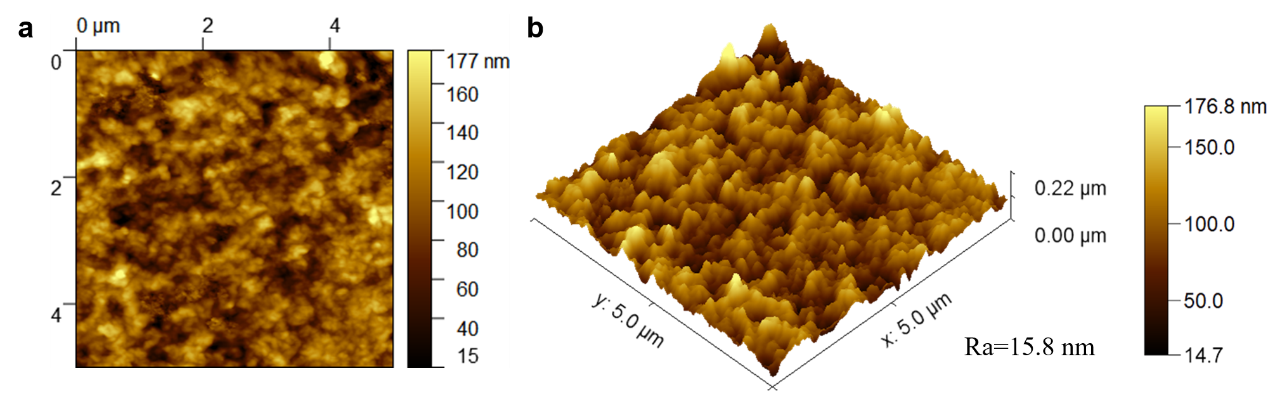


Figure S2. 2D AFM image (a) and 3D image (b) of the COF-5 film


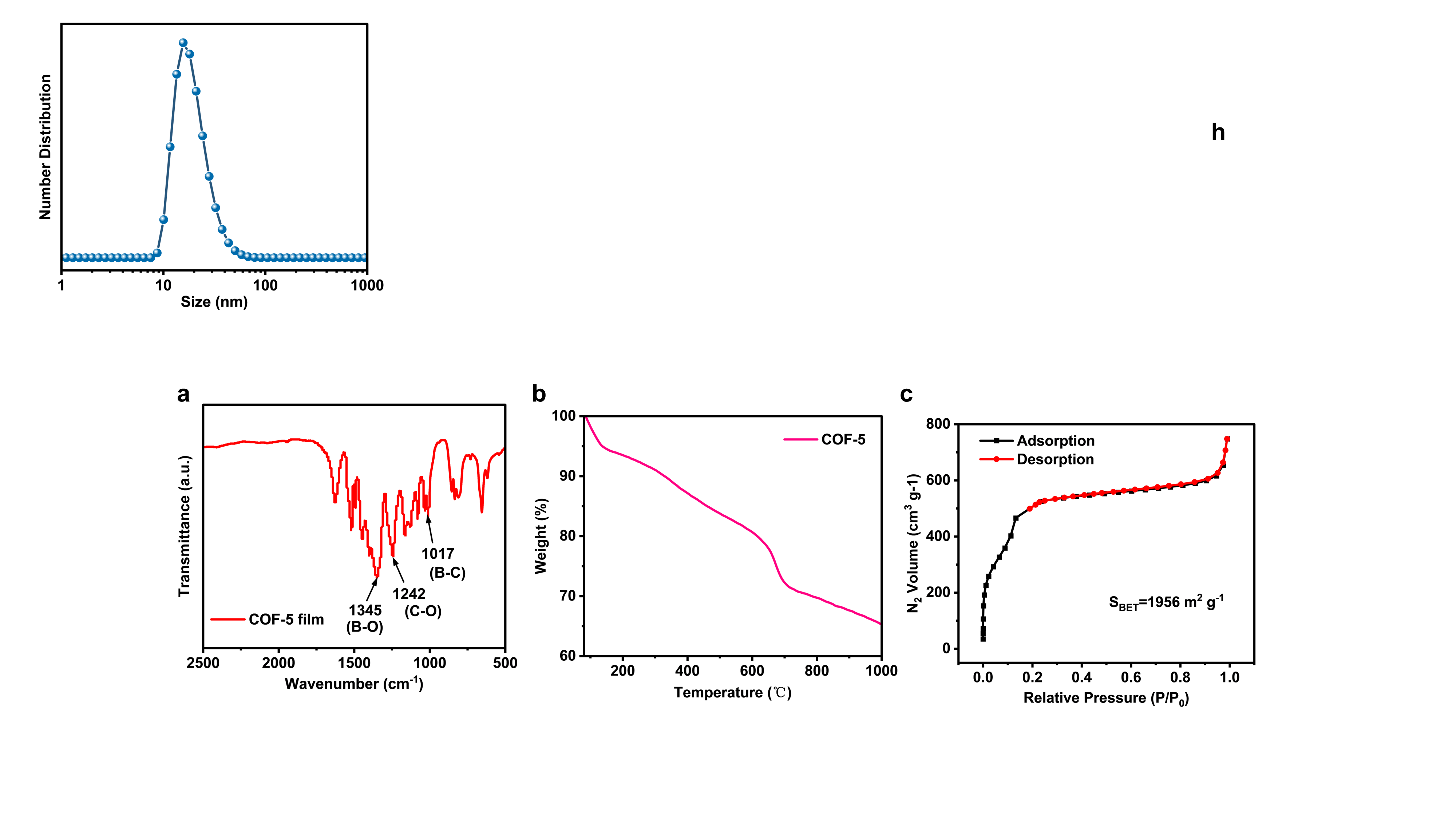


Figure S3. (a) FTIR spectrum of COF-5; (b) TGA curve of COF-5 under a nitrogen atmosphere; (c) N_2_ adsorption desorption isotherms of COF-5





Figure S4 Transient absorption results of COF-5/Si excited by 400 nm pump pulse. (a) 2D pseudocolor TA plots of COF-5/Si. (b) TA spectra of COF-5/Si at indicated time delays. (c) TA decay kinetic trace of COF-5/Si at ~721 nm.

**Table S1.** Benchmark parameters of self-powered Blue-Light photodetectors based on main 2D Materials.

| Device structure | Wavelength (nm) | Maximum *R* (mA W^-1^) | Maximum *D**  （Jones） | Rise/Decay time (ms) | Reference |
| --- | --- | --- | --- | --- | --- |
| **COF-5/Si** | **460** | **12** | **1.41×10^12^** | **0.041/0.222** | **This work** |
| Amorphous MoS_2_ | 473-2712 | 47.5 | 1.26×10^7^ | 10/16 | [1] |
| MoS_2.15_ | 445-9536 | 21800 | - | - | [2] |
| MoS_2_ | 405-650 | 164300 | 5.01×10^10^ | - | [3] |
| WSe_2_ | 370-1064 | 920 | - | 900 | [4] |
| WSe_2_ | 375-1064 | 8573 | 1.2×10^10^ | 120 | [5] |
| Bi_2_Te_3_ | 325-1550 | 74320 | 3.8×10^9^ | 420/444 | [6] |
| SnSe_2_ | 400-1200 | 2 | 2×10^11^ | 380 | [7] |
| InSe | 370-980 | 27000 | - | 0.5/1.7 | [8] |
| MoS_2_ | 454-1550 | 2300 | - | 50 | [9] |
| MoS_2_/Pattern Gallium Substrate | 460 | - | 5.6×10^8^ | - | [10] |
| Germanium/Gr | 350-1650 | 66200 | - | - | [11] |
| GaSe/MoS_2_ | 375-633 | 900 | - | 5 | [12] |
| WS_2_/Si | 400-1100 | 1100 | - | 42 | [13] |
| WS_2_/Bi_2_Te_3_ | 370-1550 | 30400 | - | 20 | [14] |
| InSe/Gr | 400-1000 | 60000 | - | 1.2 | [15] |
| Bi_2_Te_3_(TF)/pentacene | 450−3500 | 14.89 | 7.8 × 10^10^ | 1.89/2.47 | [16] |

**Supplementary equations:**

The responsivity (*R*), which represents a photodetector's ability to convert light energy into electrical energy per unit area when generating a response current under light irradiation, is defined as:^[17]^

$R=\frac{I_{ph}}{PS}$ (S1)

where *P* is the optical power density of the incident light and S is the effective area of the photodetector. *D** characterizes the capability of a photodetector to acquire a weak optical signal from a noisy signal and is defined as:^[18]^

$D^{*}=\frac{R}{\sqrt{\frac{2eI_{dark}}{S}}}$ (S2)

Where e is the electronic charge.

$\Phi=h\nu-(E_{cutoff}-E_{i})$ (S3)

where *Φ* is the work function of the COF-5 film, hυ is the incident photon energy (21.2 eV), $E_{cutoff}$is the secondary electron cutoff edge of the COF-5 film, and E_i_ is set to 0 eV.^[19]^

$E_{v}=-(\Phi+\Delta E)$ (S4)

where *ΔE* is the difference between the Fermi energy level and the maximum value of the valence band; *E*_v_ is the maximum value of the valence band; *E*_c_ is the minimum value of the conduction band.

$E_{c}=E_{v}+E_{g}$ (S5)

**Supplementary References**

[1] Z. Huang, T. Zhang, J. Liu, L. Zhang, Y. Jin, J. Wang, K. Jiang, S. Fan, Q. Li, *ACS Appl. Electron.* **2019**, *1*, 1314.

[2] Y. Xie, F. Liang, D. Wang, S. Chi, H. Yu, Z. Lin, H. Zhang, Y. Chen, J. Wang, Y. Wu, *Adv. Mater.* **2018**, *30*, 1804858.

[3] S. Ki, M. Chen, X. Liang, *J. Vac. Sci. Technol., B: Nanotechnol. Microelectron.: Mater., Process., Meas., Phenom.* **2021**, *39*, 062201.

[4] Z. Zheng, T. Zhang, J. Yao, Y. Zhang, J. Xu, G. Yang, *Nanotechnology* **2016**, *27*, 225501.

[5] J. Tai, B. Wang, D. Hu, P. Xu, Z. Zhang, *Mater. Lett.* **2021**, *287*, 129247.

[6] A. Sharma, A. Srivastava, T. Senguttuvan, S. Husale, *Sci. Rep.* **2017**, *7*, 17911.

[7] E. P. Mukhokosi, S. B. Krupanidhi, K. K. Nanda, *Sci. Rep.* **2017**, *7*, 15215.

[8] Z. Yang, W. Jie, C.-H. Mak, S. Lin, H. Lin, X. Yang, F. Yan, S. P. Lau, J. Hao, *ACS Nano* **2017**, *11*, 4225.

[9] J. Y. Wu, Y. T. Chun, S. Li, T. Zhang, J. Wang, P. K. Shrestha, D. Chu, *Adv. Mater.* **2018**, 30, 1705880.

[10] X. Liu, S. Hu, Z. Lin, X. Li, L. Song, W. Yu, Q. Wang, W. He, *ACS Appl. Mater. Interfaces* **2021**, *13*, 15820.

[11] F. Yang, H. Cong, K. Yu, L. Zhou, N. Wang, Z. Liu, C. Li, Q. Wang, B. Cheng, *ACS Appl. Mater. Interfaces* **2017**, *9*, 13422.

[12] Z. Zou, J. Liang, X. Zhang, C. Ma, P. Xu, X. Yang, Z. Zeng, X. Sun, C. Zhu, D. Liang, *ACS Nano* **2021**, *15*, 10039.

[13] R. K. Chowdhury, R. Maiti, A. Ghorai, A. Midya, S. K. Ray, *Nanoscale* **2016**, 8, 13429.

[14] J. Yao, Z. Zheng, G. Yang, *J. Mater. Chem. C* **2016**, *4*, 7831.

[15] W. Luo, Y. Cao, P. Hu, K. Cai, Q. Feng, F. Yan, T. Yan, X. Zhang, K. Wang, *Adv. Opt. Mater.* **2015**, *3*, 1418.

[16] M. Yang, J. Wang, Y. Zhao, L. He, C. Ji, X. Liu, H. Zhou, Z. Wu, X. Wang, Y. Jiang, *ACS Nano* **2018**, *13*, 755.

[17] D. Wu, M. Xu, L. Zeng, Z. Shi, Y. Tian, X. J. Li, C.-X. Shan, J. Jie, *ACS Nano* **2022**, *16*, 5545.

[18] R. Zhuo, L. Zeng, H. Yuan, D. Wu, Y. Wang, Z. Shi, T. Xu, Y. Tian, X. Li, Y. H. Tsang, *Nano research* **2019**, *12*, 183.

[19] J. Liu, Q. Hao, H. Gan, P. Li, B. Li, Y. Tu, J. Zhu, D. Qi, Y. Chai, W. Zhang, *Laser Photonics Rev* **2022**, *16*, 2200338.
